# Cosmetic Lasers in the US: Who's Using Them, the Latest Technology, and What Patients Need to Know

**DOI:** 10.1111/jocd.70235

**Published:** 2025-05-28

**Authors:** Kensington Coyle, Katarina R. Kesty

**Affiliations:** ^1^ St. Petersburg Skin and Laser Florida USA; ^2^ Kesty AI St. Petersburg Florida USA

**Keywords:** Er:YAG laser, laser surgery, laser treatment

## Abstract

**Background:**

The use of laser treatments for cosmetic and medical dermatologic conditions has increased significantly, yet provider training varies widely, raising concerns about patient safety and treatment efficacy. This study examines the availability and practice patterns of fellowship‐trained laser dermatologists and compares them to medical spas and plastic surgeons offering laser services.

**Methods:**

In September 2024, an online search and telephone survey were conducted to identify board‐certified dermatologists who completed an American Society for Dermatologic Surgery (ASDS) Cosmetics and Lasers Fellowship. Data on consultation availability, pricing, provider involvement, technology investment, and treatment customization were collected from dermatology, plastic surgery, and medical spa practices.

**Results:**

A total of 124 fellowship‐trained dermatologists were identified. Compared to medical spas and plastic surgeons, these dermatologists had longer wait times for consultations (23 vs. 4 and 11 days), higher consultation fees ($153 vs. $30 and $78), and a greater number of laser devices per practice. Physician involvement in laser procedures was significantly higher among dermatologists (60%) compared to plastic surgeons (33%) and medical spas (9%). Medical spas relied heavily on nonphysician providers (26%) and laser technicians (56%), with only 41% providing direct on‐site supervision. The majority (98%) of dermatologists and plastic surgeons customized laser treatments, compared to 63% of medical spas. Dermatologists dedicated more clinical time to laser procedures, with 19% spending over 50% of their practice on lasers, compared to none at medical spas and plastic surgery offices.

**Conclusion:**

Fellowship‐trained laser dermatologists provide more direct physician involvement, greater technological resources, and highly customized treatments compared to medical spas and plastic surgeons. While medical spas offer lower costs and shorter wait times, the lack of physician oversight and limited training among providers may represent substandard care. These findings highlight the importance of provider qualifications in ensuring optimal patient outcomes and underscore the value of specialized laser training in dermatology.

## 1 Introduction

Laser treatments for cosmetic and medical dermatologic conditions have become increasingly popular in recent years, with a growing number of providers offering these services [1]. While it is well‐established in medicine that higher procedural volumes are associated with improved patient outcomes, the level of training among laser operators in the United States varies significantly [2]. Providers range from Fellowship‐Trained Laser Surgeons, who undergo over a decade of comprehensive medical and procedural education, to laser technicians who may complete only 1–2 weeks of training after high school before using lasers and energy‐based devices. Such variability raises concerns regarding patient safety and treatment efficacy, especially given the powerful and potentially harmful nature of laser devices when used improperly [3, 4, 5, 6, 7].

State regulations governing the use of cosmetic lasers are inconsistent, with licensing and training requirements differing across jurisdictions [8]. Despite these regulatory gaps, patient awareness surrounding provider qualifications has improved, driven in part by the widespread dissemination of information through social media and online platforms. Patients are becoming increasingly proactive in advocating for high‐quality medical care, seeking out physicians with advanced training in cosmetic and laser procedures. Indeed, data from laser‐related litigation between 2012 and 2020 indicate that dermatologists were only involved in 3% of litigated cases, while nonphysician operators demonstrated significantly higher complication rates [9].

To better understand the landscape of laser providers and the accessibility of physician‐led care, this study investigates the availability and practice patterns of dermatologists who have completed a Cosmetics and Lasers Fellowship through the American Society for Dermatologic Surgery (ASDS), the only formal education that exists for Lasers. In addition, the study examines the presence of nearby medical spas and plastic surgeons offering similar services. By evaluating the differences in provider types and their respective practice environments, this research aims to shed light on the variability in laser care delivery across the United States and highlights the importance of specialized training in ensuring patient safety and optimal outcomes.

## 2 Methods

In September 2024, the authors conducted an online search to identify dermatologists who completed a Cosmetics and Lasers Fellowship through the American Society for Dermatologic Surgery (ASDS) (hereafter referred to as “Dermatologist(s)”). The search utilized various terms, including “Dermatologist Laser,” “Laser Surgeon,” “Cosmetics Fellowship,” “Cosmetic Dermatologist,” and “Fellowship‐Trained Dermatologist.” In addition to online search engines, the ASDS website and directory were used to identify qualifying dermatologists.

A standardized script, developed by the authors, was used to inquire about new patient services. Using this script, trained secret shoppers contacted Dermatology practices via telephone and documented the responses provided by staff members.

Dermatologists were excluded from the study if they were retired, nonpracticing, did not perform laser procedures (as indicated on their website or by staff during telephone inquiries), or practiced outside the United States. For each Dermatologist who completed an ASDS Cosmetics and Lasers Fellowship, the primary office location was confirmed, including the state and zip code. This geographic data was then used to identify medical spas and plastic surgeons offering laser procedures in the same or neighboring zip codes. To locate these practices, search engines were used with terms such as “Med Spa + zip code” and “Plastic Surgeon + zip code” (e.g., “Med Spa + 21268”). One designated research team member was responsible for identifying practices in close proximity to the dermatologists' offices.

Telephone calls to the identified offices were conducted over a 30‐day period to collect data regarding the use of lasers within these practices. All collected data were organized and recorded using Microsoft Excel (Excel 2021).

This study did not involve experimentation on human subjects and was therefore exempt from Institutional Review Board (IRB) review. The authors were responsible for conducting database searches, data collection, review, and analysis.

## 3 Results

A total of 124 dermatologists who completed a Cosmetics and Lasers Fellowship through the American Society for Dermatologic Surgery were included in this study. An equal number of medical spas and plastic surgery offices were analyzed for comparison.

Most dermatologists (121; 97.5%) were accepting new patients, while three (2.5%) were not. All medical spas and plastic surgery offices surveyed were accepting new patients. The average wait time for a new patient consultation was 23 days for dermatologists, 4 days for medical spas, and 11 days for plastic surgeons.

**FIGURE 1:**
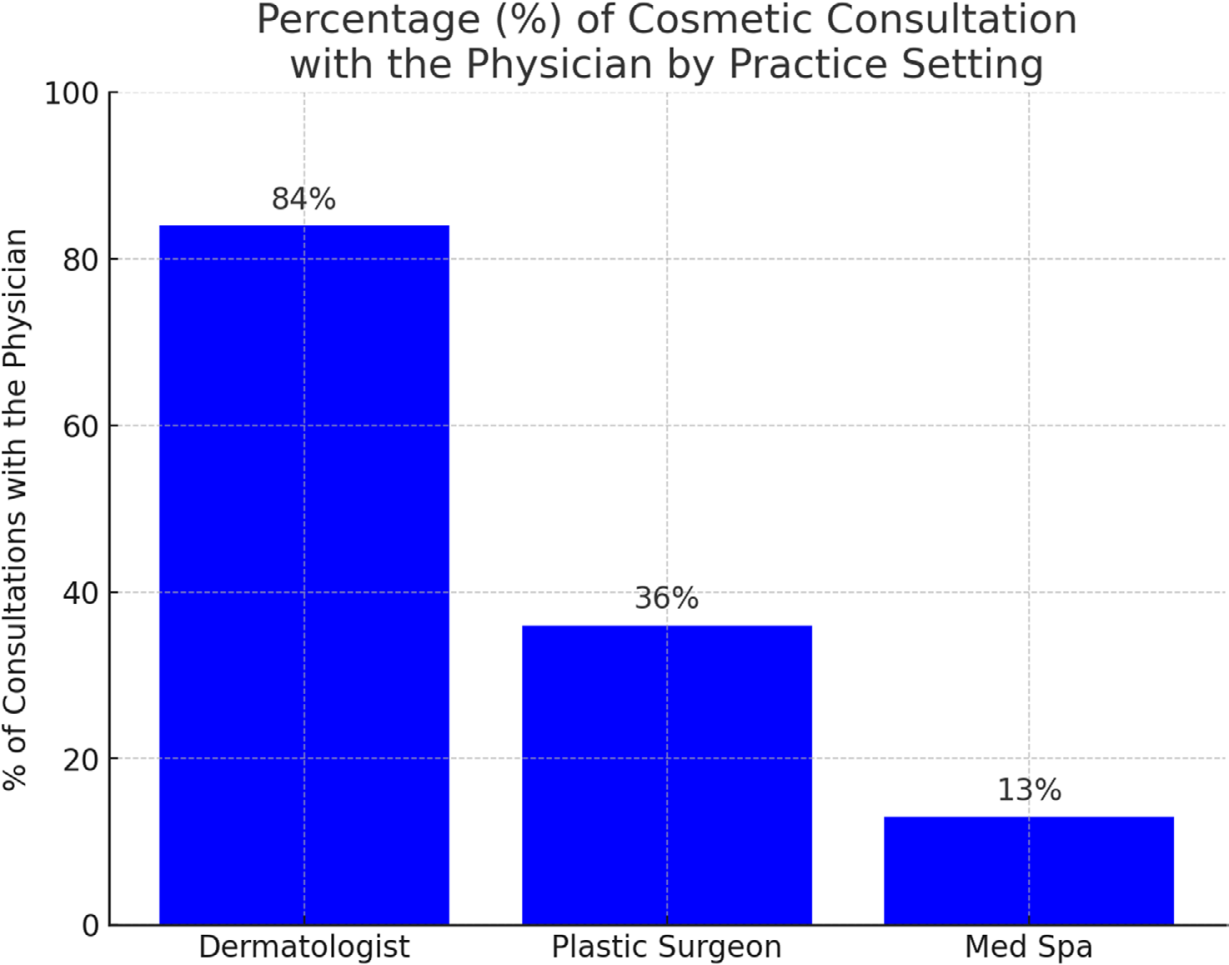
Percentage (%) of cosmetic consultations that are with the physician, by practice setting.

The mean cost for an initial consultation was $153 for dermatologists, $78 for plastic surgeons, and $30 for medical spas. In dermatology offices, consultations were conducted directly with the physician 84% of the time, compared to 13% of medical spas and 36% of plastic surgery offices (Figure 1).

Dermatology practices employed an average of 11 physicians, while medical spas averaged one physician and plastic surgery offices averaged two physicians.

**FIGURE 2:**
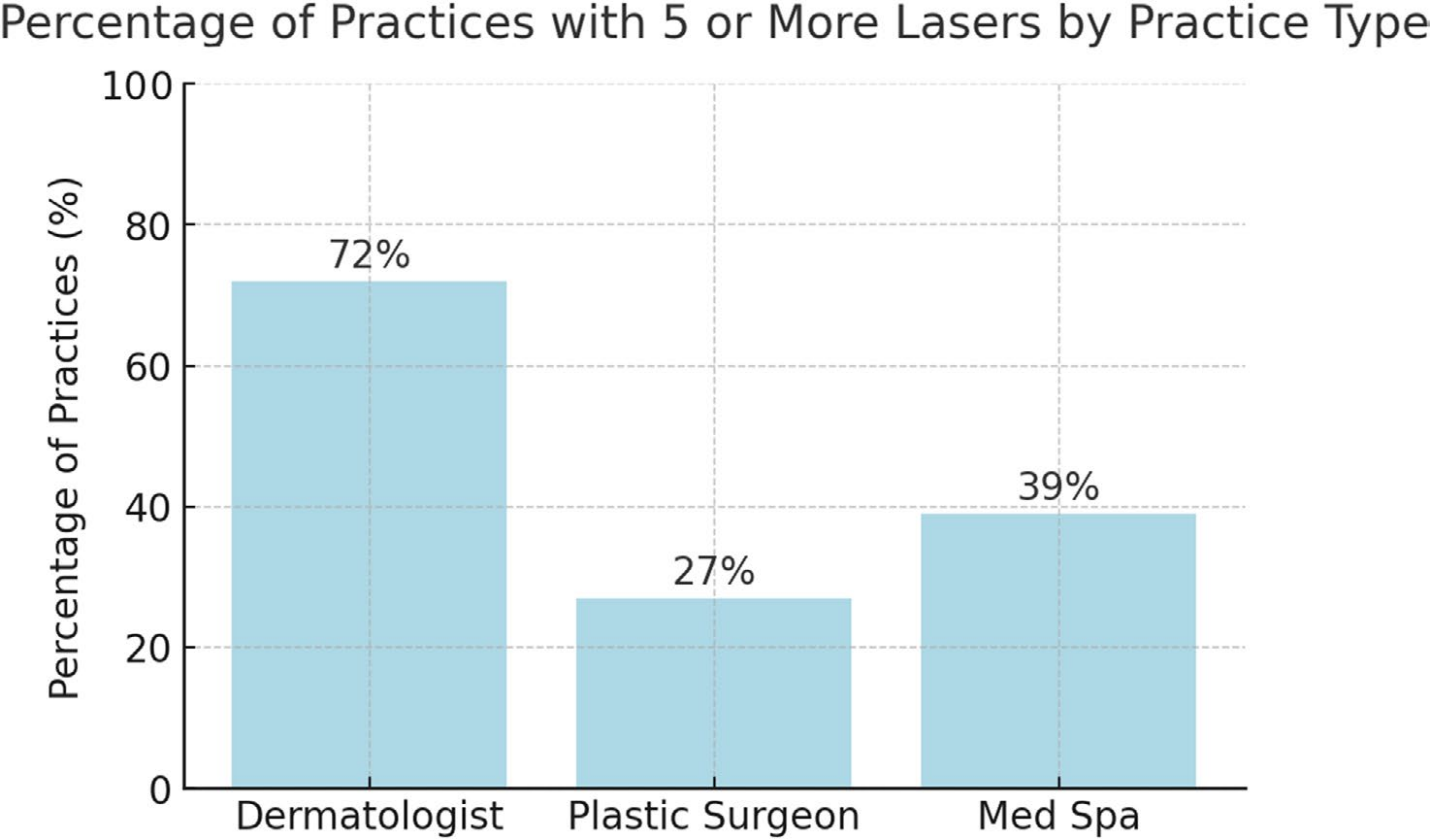
Percentage (%) of practices with five or more lasers, grouped by practice type.

The number of lasers and energy‐based devices per practice varied. Among dermatologists, 18% of practices had 21 or more devices, 15% had between 10 and 20 devices, 39% had between 5 and 10 devices, 20% had between 2 and 4 devices, and 3% had only one device (Figure 2). Among medical spas, 2% had 21 or more devices, 6% had between 10 and 20 devices, 19% had between 5 and 10 devices, 59% had between 2 and 4 devices, and 7% had a single device. Plastic surgery offices reported 2% with 21 or more devices, 8% with between 10 and 20 devices, 29% with between 5 and 10 devices, 41% with between 2 and 4 devices, and 18% with one device.

The average cost of an ablative laser procedure was $4055 in dermatology offices, $3154 in plastic surgery offices, and $1157 in medical spas. For nonablative laser treatments, the average cost was $1083 for dermatologists, $736 for plastic surgeons, and $485 for medical spas. 98% of dermatologists customize the Laser combination and settings for each patient, compared to 63% of Medical Spas and 98% of Plastic Surgeons.

**FIGURE 3:**
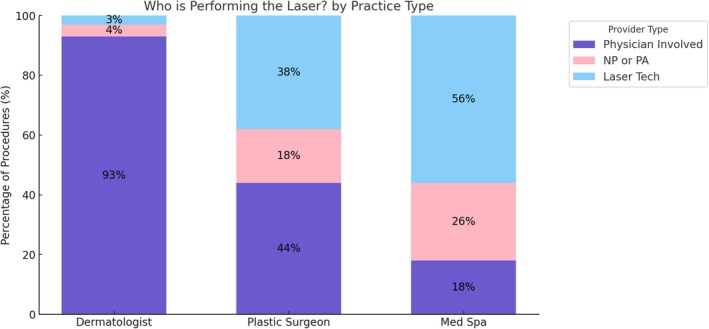
What type of provider is performing the laser treatment—physician, nurse practitioner or physician assistant, or laser technician.

Physicians performed laser procedures in 60% of dermatology offices, 33% of plastic surgery offices, and 9% of medical spas. Nonphysician providers (NPPs) performed laser treatments in 4% of dermatology offices, 18% of plastic surgery offices, and 26% of medical spas. Laser technicians performed procedures in 3% of dermatology offices, 35% of plastic surgery offices, and 56% of medical spas. A combination of providers performed laser procedures in 25% of dermatology offices, 11% of plastic surgery offices, and 12% of medical spas (Figure 3).

**FIGURE 4:**
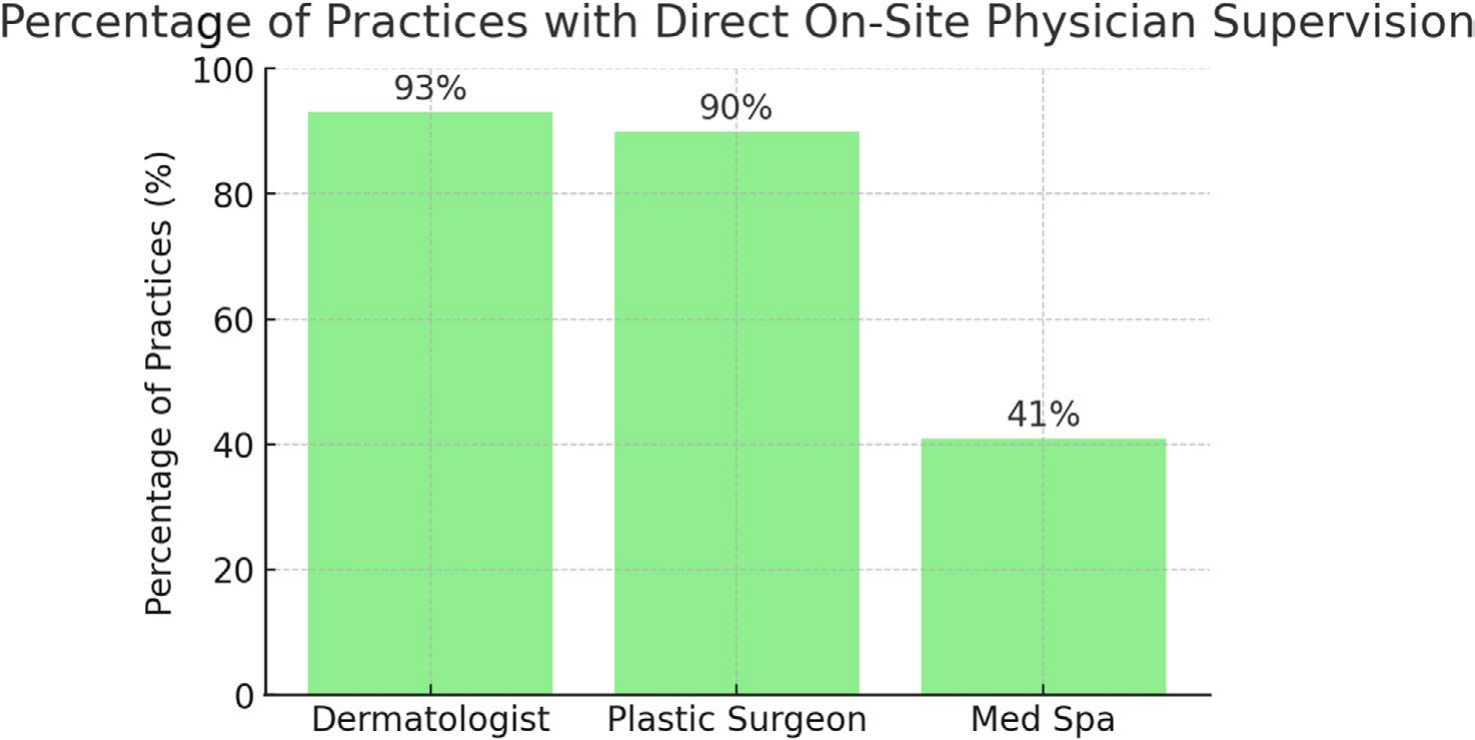
The percentage (%) of practices with direct on‐site physician supervision by practice type.

Direct, on‐site supervision of NPPs or laser technicians was present in 93% of dermatology offices, 90% of plastic surgery offices, and 41% of medical spas (Figure 4).

**FIGURE 5:**
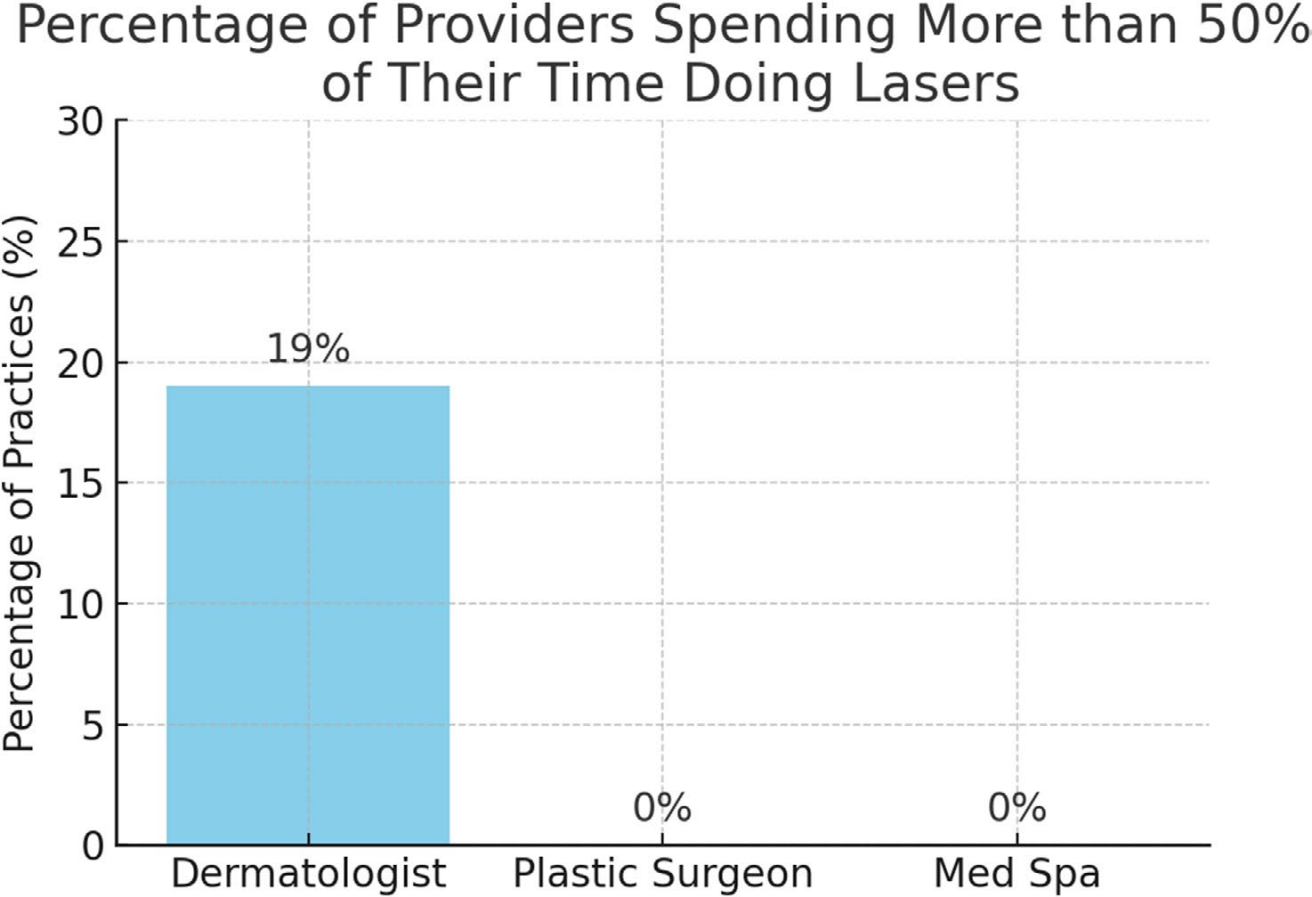
The percentage of providers, by type, that spend more than 50% of their practice time doing lasers.

The percentage of clinical time dedicated to laser procedures varied. Among dermatologists, 19% spent more than 50% of their clinical time on laser treatments, 34% spent between 25% and 50%, 23% spent between 1% and 25%, and 10% spent less than 1%. No medical spa or plastic surgery providers reported spending more than 50% of their clinical time on laser procedures. At medical spas, 25% of providers spent between 25% and 50% of their time on laser treatments, 29% spent between 1% and 25%, and 41% spent less than 1%. Among plastic surgeons, 15% spent between 25% and 50% of their clinical time on lasers, 56% spent between 1% and 25%, and 25% spent less than 1% (Figure 5).

These results highlight notable differences in patient access, treatment costs, provider involvement, technology availability, and time allocated to laser treatments across dermatology, medical spa, and plastic surgery practices.

## 4 Discussion

This study highlights significant differences in patient access, provider training, practice characteristics, and pricing among dermatologists who completed a Cosmetics and Lasers Fellowship through the American Society for Dermatologic Surgery (ASDS), medical spas, and plastic surgery practices offering laser services. These differences have important implications for patient safety, quality of care, and informed decision‐making.

**FIGURE 6:**
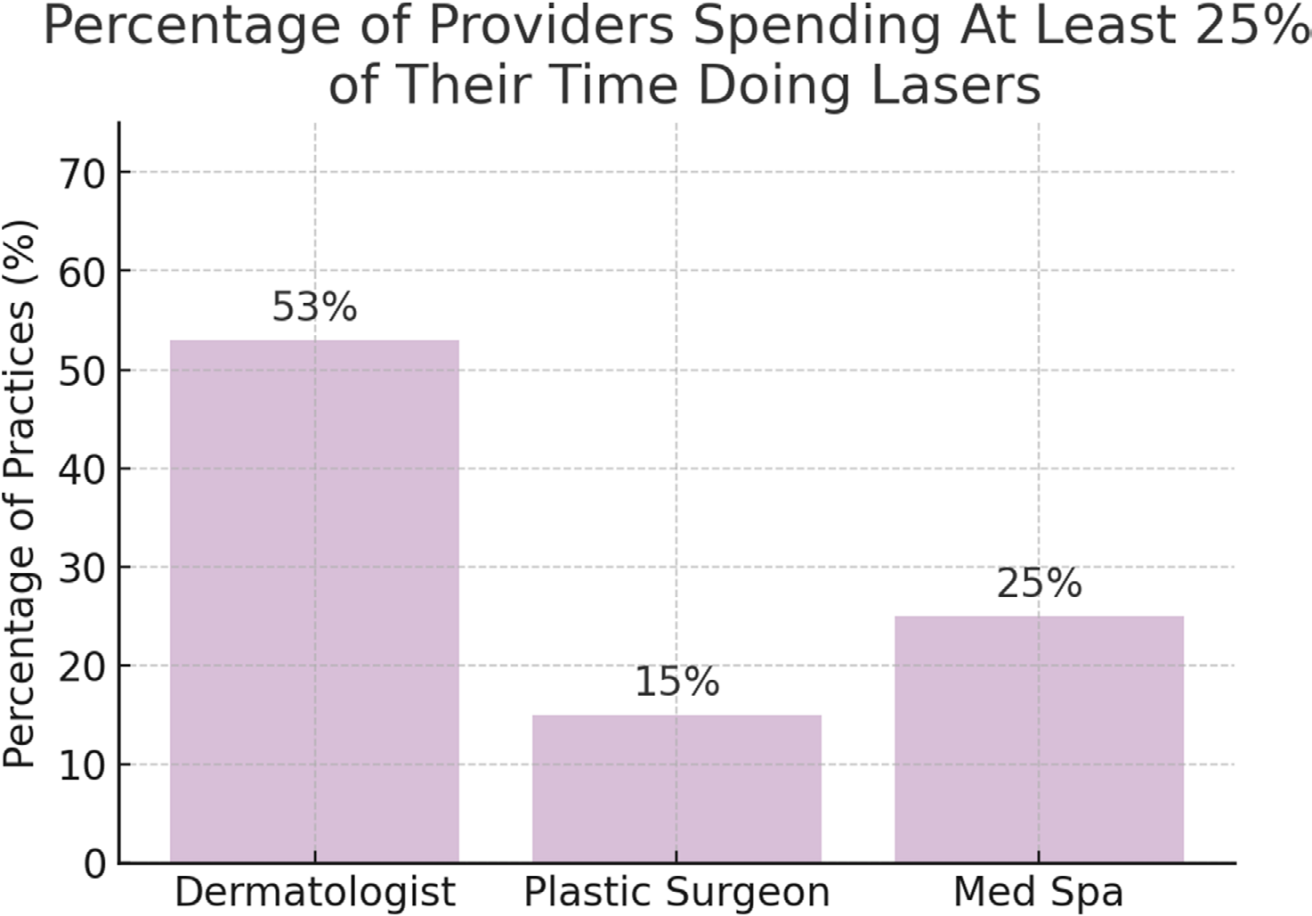
Figure 5: The percentage of providers by type that spend more than 25% of their practice time doing lasers.

**FIGURE 7:**
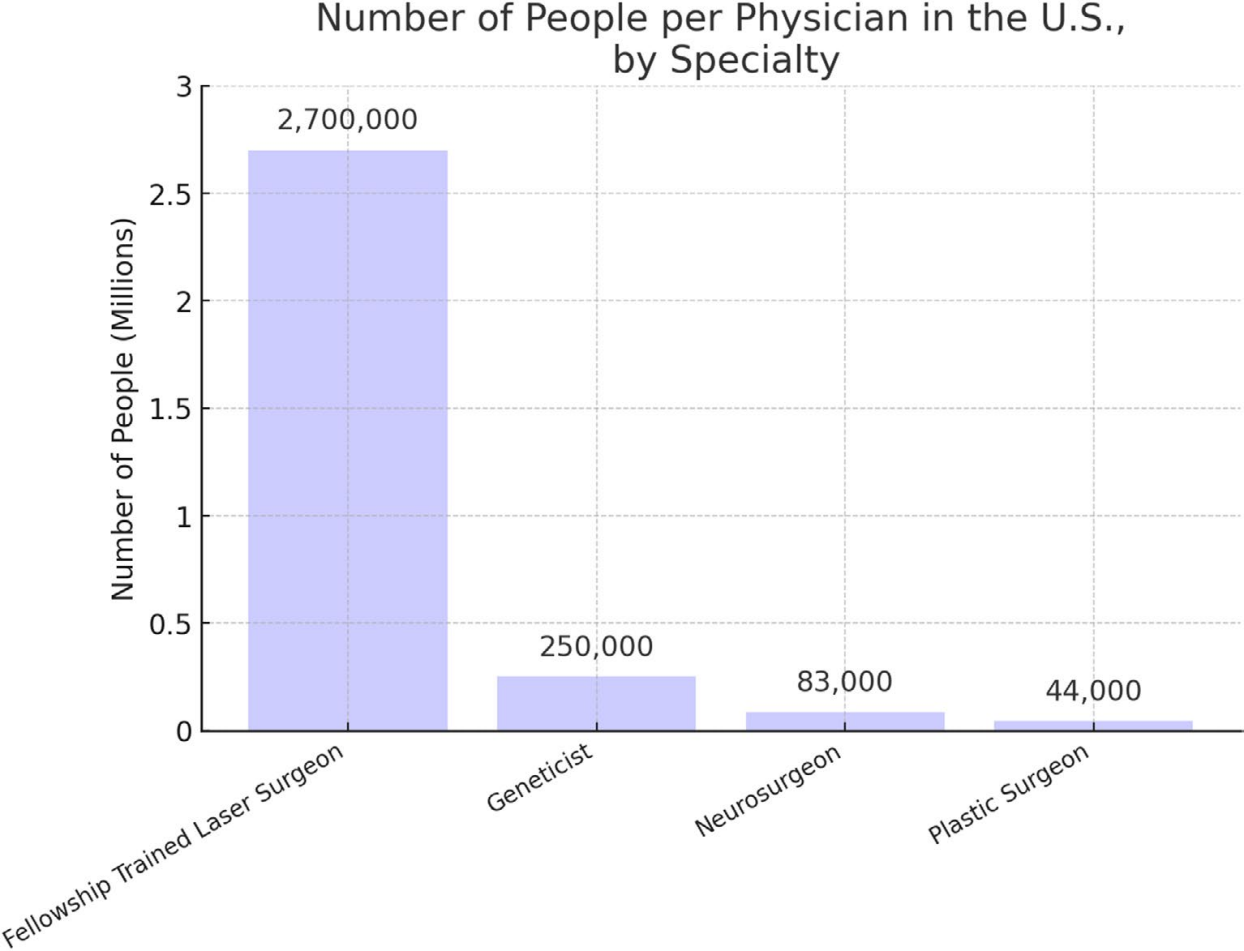
The number of people per type of Physician in the United States.

Laser fellowship‐trained dermatologists are extremely rare in the United States. With a population of approximately 333 million people and only 124 laser fellowship‐trained dermatologists, there is roughly one laser surgeon for every 2.7 million people (Figure 6). In comparison, there are approximately 4000 neurosurgeons in the United States, equating to one neurosurgeon for every 83 000 people [10]. Similarly, there are about 1240 geneticists, which corresponds to one geneticist for every 250 000 people [11]. By contrast, there are approximately 7725 plastic surgeons, equating to one plastic surgeon for every 44 000 people [12]. These figures highlight the extreme rarity of laser fellowship‐trained dermatologists relative to other medical specialists (Figure 7).

While the vast majority of dermatologists (97.5%) were accepting new patients, their average wait time for a consultation (23 days) was notably longer compared to medical spas (4 days) and plastic surgeons (11 days). The longer wait times in dermatology offices may reflect higher patient demand and a limited supply of highly specialized providers.

Consultation costs were highest among dermatologists, averaging $153, compared to $30 at medical spas and $78 at plastic surgery practices. Although the higher price point for Dermatology consultations may be a deterrent for some patients, it likely reflects the added value of physician expertise, comprehensive evaluation, and individualized treatment planning. This is supported by the finding that consultations were conducted by physicians at 84% of dermatology offices versus only 13% at medical spas and 36% at plastic surgery offices.

Dermatology practices demonstrated significantly greater resources and technological investments in laser and energy‐based devices. Nearly one‐fifth (18%) of dermatologists had more than 21 lasers, compared to just 2% of medical spas and plastic surgery offices. This extensive array of devices allows for greater versatility in tailoring treatments to individual patient needs. In contrast, the majority of medical spas (59%) and a substantial proportion of plastic surgeons (41%) operated with just 2–4 lasers, potentially limiting treatment options.

Dermatology practices also had larger teams of physicians (average of 11), suggesting a more collaborative environment and access to a broader range of expertise. By comparison, medical spas and plastic surgery offices averaged one and two physicians, respectively.

Provider qualifications and direct involvement in laser procedures varied markedly. In Dermatology practices, 60% of laser treatments were performed by physicians, with nonphysician providers (NPPs) and laser technicians involved in a minority of cases (4% and 3%, respectively). Conversely, medical spas relied heavily on laser technicians (56%) and NPPs (26%) to perform procedures, with physician involvement limited to 9% of laser cases. Plastic surgeons' offices fell between these two extremes, with 33% of lasers performed by physicians and 35% by laser technicians.

The high physician involvement in dermatology offices, coupled with a 93% rate of on‐site supervision of nonphysician providers, underscores a commitment to patient safety and procedural oversight. Medical spas, in contrast, reported on‐site supervision in only 41% of cases, raising concerns about the adequacy of safety protocols in these settings.

Customization of laser treatments was highest among dermatologists and plastic surgeons (98% each), compared to 63% at medical spas. This difference suggests that physician‐led practices are more likely to tailor treatments based on individual patient characteristics, which leads to improved outcomes and reduced complication risks.

Costs of laser procedures reflected both provider expertise and practice resources. Ablative laser treatments were most expensive at dermatology offices ($4055), followed by plastic surgeons ($3154) and medical spas ($1157). Similar trends were observed for nonablative procedures. The higher costs associated with dermatologists and plastic surgeons likely reflect the use of advanced technology, comprehensive pre and posttreatment care, and the direct involvement of highly trained physicians.

dermatologists demonstrated a stronger focus on laser treatments within their clinical practice. Over half (53%) of dermatologists spent at least 25% of their clinical time performing laser procedures, compared to 25% of medical spas and 15% of plastic surgeons. Notably, 19% of dermatologists dedicated more than 50% of their clinical time to lasers, underscoring their specialization in this field. By contrast, no medical spa or plastic surgery providers reported spending more than half their time on laser treatments, which may impact procedural volume, expertise, and patient outcomes.

The findings of this study align with previous research indicating that higher procedural volumes and advanced training correlate with improved patient outcomes. dermatologists with fellowship training in lasers not only invest more in technology but also provide more direct physician involvement and customized care. These factors are critical in reducing complications and achieving optimal results. In contrast, medical spas—despite offering quicker appointments and lower costs—often rely on less‐trained providers and have less robust oversight, potentially providing substandard care.

### 4.1 Limitations

This study relied on information provided by practice staff during telephone inquiries, which may be subject to inaccuracies or inconsistencies. This study is based on information obtained through telephone interviews with practice staff, which may be subject to reporting inaccuracies or inconsistencies. Additionally, physicians and licensed providers were not interviewed directly; therefore, the data reflect the knowledge and experience of the staff member who responded to the inquiry. Additionally, the scope was limited to providers practicing in the United States, and findings may not be generalizable to other countries. The terms ablative and nonablative laser can refer to different procedures and various types of lasers, depending on the provider. As a result, pricing for these treatments may not offer a direct comparison between practices. Future research could explore patient outcomes and complication rates in greater detail to further elucidate the impact of provider type and training on laser treatment efficacy.

## 5 Conclusion

This study highlights significant differences in access, cost, provider involvement, technology availability, and treatment customization among fellowship‐trained dermatologists, medical spas, and plastic surgery practices offering laser procedures. While medical spas and plastic surgeons often provide faster appointment availability and lower consultation costs, fellowship‐trained dermatologists demonstrate a higher level of physician involvement, advanced technological resources, and more individualized treatment plans. These distinctions are essential for patients seeking quality, effective, and tailored laser treatments. Patients should carefully consider provider qualifications, practice resources, and the level of physician oversight to ensure optimal safety and long‐term outcomes. Investing in care from fellowship‐trained dermatologists can provide enhanced precision, personalized treatment approaches, and higher standards of patient care, ultimately leading to better overall results.

## Author Contributions

K.R.K. and K.C. conceived the study and wrote and revised the manuscript. All authors have reviewed and approved the article for submission.

## Conflicts of Interest

The authors declare no conflicts of interest.

## Data Availability

The data that support the findings of this study are available from the corresponding author upon reasonable request.
